# Glucagon-like peptide-1 agonists’ effects on glycemic control, weight loss, and beta cells function in type 1 diabetes

**DOI:** 10.3389/fendo.2026.1758755

**Published:** 2026-03-11

**Authors:** Hyder O. Mirghani, Laila Albishi, Sawsan Mohmed Alblewi

**Affiliations:** 1 Internal Medicine Department, Faculty of Medicine, University of Tabuk, Tabuk, Saudi Arabia; 2 Pediatrics Department, Faculty of Medicine, University of Tabuk, Tabuk, Saudi Arabia

**Keywords:** GLP-1 agonists, HbA1c, T1DM, weight, β cells

## Abstract

**Background:**

Insulin is an effective treatment for type 1 diabetes mellitus (T1DM), and a significant proportion of patients are not controlled, develop hypoglycemia, and gain weight. Therefore, adjuvant therapies to mitigate the above are highly needed. Meta-analyses on the effect of glucagon peptide agonists (GLP-1 agonists) on weight loss and HbA1c are scarce. We aimed to assess the effects of GLP-1 agonists on HbA1c, weight, and C-peptide in patients with T1DM with obesity/overweight and normal weight.

**Methods:**

We searched PubMed, Web of Science, Cochrane Library, and Google Scholar from inception up to October 30, 2025. The keywords T1DM, GLP-1 agonists, weight, HbA1c, hyperglycemia, adverse effects, hypoglycemia, time in the range, continuous monitoring, blood glucose, C-peptide, and complications were used. We identified 904 studies; from them, 33 full texts were eligible, and 18 studies were included in the meta-analysis.

**Results:**

GLP-1 agonists achieved a higher reduction in weight, HbA1c, and time spent in hyperglycemia compared to controls, MD=-4.28, 95% *CI*, -5.06--3.49, MD=-0.4, 95% *CI*, -0.77--0.03, and MD=-1.98, 95% *CI*, -3.68--0.28, respectively. The time spent in hypoglycemia, MD = 0.08, 95% *CI*, -0.88-1.04, and the maximum stimulated C-peptide were not different. The total adverse events were higher in GLP-1 agonists.

**Conclusion:**

GLP-1 agonists reduced weight, HbA1c, and time in hyperglycemia significantly compared to controls at the cost of total side effects. The stimulated C-peptide and hypoglycemia were not different between the two groups; further well-controlled trials investigating the role of GLP-1 agonists in newly diagnosed, and normal body weight T1DM are recommended.

## Introduction

Type 1 diabetes results from autoimmune destruction of the β cells of the pancreas, leading to severe insulin deficiency. The disease constitutes 5-10% of diabetes, and the disease is rising as a significant clinical and public health burden globally (1). Insulin deficiency necessitates lifelong insulin therapy to prevent ketoacidosis and death. Uncontrolled hyperglycemia leads to severe complications that negatively impact the patient’s quality of life and reduce life expectancy (2).

Unlike type 2 diabetes mellitus, for which many drug choices are available, the only available therapy for type 1 diabetes is insulin (Sodium Glucose cotransporters inhibitors are used as adjuvant therapy for uncontrolled T1DM and a body mass index (BMI) of ≥27 kg/m^2^). Despite the great advances in insulin, insulin delivery, and glucose monitoring. However, a significant number of patients with type 1 diabetes develop serious complications due to poor glycemic control (3–5). Importantly, only a minority of type 1 diabetes patients are approaching American Diabetes Association glycemic targets (6). Nearly half of type 1 diabetes cases are diagnosed in the adult population, and an overlap with type 2 diabetes was observed in 10% of patients (7, 8).

Another important feature of type 1 diabetes is the presence of insulin resistance. Insulin resistance in T1DM is more prevalent in the liver and skeletal muscles (tissue-specific). Left ventricular hypertrophy, diastolic dysfunction, and altered oxygen uptake are suggested mechanisms (9–11). Subcutaneous insulin could lead to hepatic hypoinsulinemia, hepatic glucose synthesis, and a rise in growth hormone and Insulin like growth factor-binding proteins (12, 13).

Autoimmunity with continuous beta-cell (β-cell) destruction is observed long before the symptoms of diabetes, and three stages of T1DM were recognized (stage 1 with autoimmunity, normal glucose, and no symptoms, stage 2 in which a symptomatic dysglycemia developed, and stage 3 with symptoms and dysglycemia) (14).

Because of the above, and the fact that obesity is common among patients with type 1 diabetes, Merger et al. (15) reported that 25% of patients with type 1 diabetes have features of metabolic syndrome. Therefore, glucagon-like peptide-1-receptor agonists (GLP-1 agonists) are attractive adjuvant therapy in type 1 diabetes due to their weight reduction dimension, and glycemic benefits (16). Glucagon-like peptide-1 receptor agonists are secreted from the gut neuroendocrine cells and exert their glycemic and weight reduction effects by inhibiting glucagon release, augmenting insulin release, satiety, and decreasing gastric emptying (17). Animal studies showed that GLP-1 agonists increase β-cell proliferation, and decrease their death, resulting in expansion of beta cell mass, and a small piece of evidence is present for improving β-cell function in type 2 diabetes (18, 19). The role of GLP-1 in beta cell preservation is controversial. Ahrén et al. (20) and Mathieu et al. (21) observed a modest improvement in HbA1c, decreased insulin dose, and weight reduction by liraglutide. However, the authors observed higher rates of hypoglycemia, ketoacidosis, and gastrointestinal adverse events.

GLP-1 agonists were approved for the treatment of obesity in 2009; the weight reduction is mainly through reduction of food intake, with minimal effects on fat oxidation (22). Many new long-acting preparations are available for the treatment of obesity and type 2 diabetes, and oral semaglutide is now available (18).

GLP-1 agonists are attractive because they address the drawbacks of insulin (weight gain, hypoglycemia, and the need for daily injection). The delayed gastric emptying observed in GLP-1 agonists is attractive for postprandial blood glucose regulation and weight reduction in type 1 diabetes, but the ketosis and hypoglycemia observed result in the lack of their approval by the Food and Drug Administration (22, 23). Nevertheless, there is growing off-label use of GLP-1 agonists for type 1 diabetes (24). GLP-1 agonists could significantly change the management of type 1 diabetes by β-cell preservation and improving glycemic parameters with lower insulin doses; this hopeful path is particularly applicable in newly diagnosed type 1 diabetic patients (25). Another attractive feature of GLP-1 agonists addresses the inappropriate glucagon release by α cells of the pancreas, resulting in glycemic control with lower rates of hypoglycemia (26).

The only real possible cure for type 1 diabetes is promoting β-cell replication and survival to prevent loss of β-cell mass. Therefore, GLP-1 agonists are an attractive adjuvant treatment for T1DM (27). GLP-1 agonists bind to the receptors in β cells of the pancreas, improve their function by preservation, decrease apoptosis, and promote regeneration (28–30).

Meta-analyses on the effects of GLP-1 agonists on weight loss and glycemic regulation are scarce. Previous meta-analyses on the effects of GLP-1 agonists on glycemic parameters and weight reduction showed contradicting results and are limited by the small number of included studies (31), including studies with overlap (published by the same authors), and high heterogeneity (32, 33). Importantly, many studies have been published on this important topic since the publication of some meta-analyses (34). Therefore, this meta-analysis assesses the effects of GLP-1 agonists on HbA1c and weight in patients with T1DM with obesity/overweight, and normal body mass index. In addition, we assessed the effect of GLP-1 agonists on C-peptide and the differences in HbA1c in C-peptide+ve and C-peptide –ve patients.

## Methods

### Eligibility criteria according to PICOS

This study was conducted in September and October 2025 to assess the effects of GLP-1 agonists on weight and HbA1c in patients with T1DM with obesity/overweight, and normal weight.

### Inclusion criteria

We included randomized controlled trials, prospective and retrospective studies, case-control, and interventional studies from inception up to October 2024. The studies must compare the effects of GLP-1 agonists and placebo on body weight and HbA1c at baseline and following GLP-1 agonists in patients with T1DM exclusively. Studies that reported total adverse events, hypoglycemia, hyperglycemia, time spent in hypoglycemia, and time spent in hyperglycemia were included.

### Exclusion criteria

Case reports, case series, posters, experts’ opinions, editorials, commentaries, protocols without results, and reviews were excluded.

### Outcome measures

#### The outcome measures were

The effects of GLP-1 agonists on weight, HbA1c, and C-peptide level. In addition, total side events, hypoglycemia, hyperglycemia, time spent in hypoglycemia, and time spent in hyperglycemia were investigated.

#### Time spent in hypoglycemia and hyperglycemia

Time spent in hypoglycemia and hyperglycemia was defined as blood glucose below 70mg/dl and above 180mg/dl, respectively.

#### Literature search and data extraction

**Figure 1:**
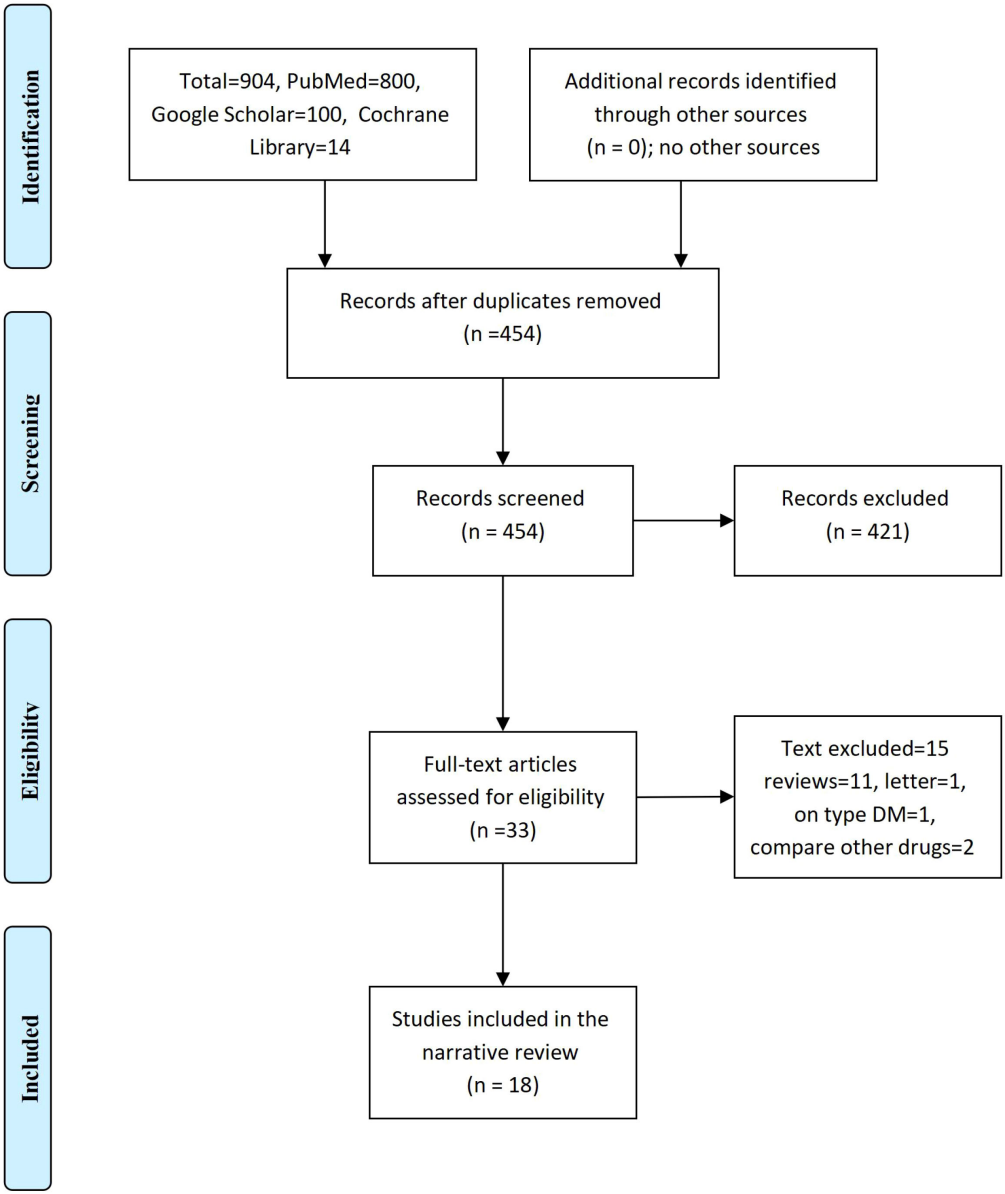
The effects of GLP-1 agonists on weight reduction and glycemic indices in patients with type 1 diabetes (The PRISMA Chart).

We searched PubMed MEDLINE, Web of Science, Cochrane Library, and Google Scholar from the first published article up to June 30, 2025. The keywords include T1DM, GLP-1 agonists, HbA1c, weight, glycated hemoglobin, hypoglycemia, adverse effects, side effects, time in the range, continuous monitoring, blood glucose, C-peptide, and diabetes complications. The titles and abstracts of the retrieved articles and the full text references were searched for relevant articles. We identified 904 studies and 454 remain after the removal of duplication; from them, 33 full texts were eligible, and only 18 studies were included in the final meta-analysis **Figure 1**.

### Data extraction

The first author’s name, year and country of publication, age of the participants, females%, study type, number of participants, the study duration, duration of diabetes, body mass index, type of GLP-1 agonist, total side effects, number of hyper and hypoglycemia, time spent in hypoglycemia, time spent in hyperglycemia, and C-peptibe level we recorded in excel sheet. All the outcomes were reported before and after GLP-1 agonists (**Tables 1**–**3**).

**Table 1:** Basic characteristics of the study group.

Author	Country	Study type	BMI/kg/m², GLP-1 agonists/control	Females %, GLP-1 agonists/control	Age years, GLP-1 agonists/control	Duration of diabetes years, GLP-1 agonists/control	Time spent in hypoglycemia, GLP-1 agonists/control	Time spent in hyperglycemia, GLP-1 agonists/control
Aherin et al., 2016 (20)	USA	Trial	43.3 vs. 42.7	54% vs. 54%	28.9 ± 0.06 28.9 ± 0.07	21.2 vs. 20.7	Not assessed	Not assessed
Mathieu et al., 2016 (21)	Denmark	Trial	43.4 ± 12.6 vs. 43.7 ± 13	52.2% vs. 51.9%	29.43 ± 5.2 Vs. 29.8 ± 5.6	21.3 ± 12.3 vs. 21.6 ± 1.8	Not assessed	Not assessed
Al-Ozairi et al., 2023 (37)	Kuwait	Retrospective	36.5 ± 9.2 vs. 33.8 ± 9.0	62.2% vs. 47.5%	31.3 ± 3.7 vs. 30.1 ± 2.9	18.7 ± 8.6 vs. 18.7 ± 10.2	Not assessed	Not assessed
Dejgaard et al., 2016 (38)	Denmark	Trial	30.3 ± 3.5 vs. 29.8 ± 3.1	60% vs. 70%	47 ± 13 vs. 49 ± 12	20 ± 12 vs. 25 ± 12	1.2 ± 0.9 vs. 1.1 ± 0.45	13.4 ± 1.45 vs. 12.8 ± 1.3
Dejgaard et al., 2019 (39)	Denmark	Trial	30 ± 2 vs. 29 ± 3	68% vs. 68%	50 ± 14 vs. 43 ± 12	21 ± 9.5 vs. 20 ± 10	Not assessed	Not assessed
Dejgaard et al., 2024 (40)	Denmark	Trial	27 vs. 29	29% vs. 44%	24 vs. 23	4.2 ± 1.3 vs. 4.5 ± 1.2	Not assessed	Not assessed
Fandsen et al., 2015 (41)	Denmark	Trial	39.5 ± 2.7 vs. 36.1 ± 1.6	39% vs. 28%	Not assessed.	18.33 ± 2.0 vs. 19.56 ± 1.6	Not assessed	Not assessed
Ghanim et al., 2020 (42)	Italy	Trial	Not assessed	43.3%	22.5	Not assessed	Not assessed	Not assessed
Grag et al., 2025 (43)	USA	Retrospective	41 ± 11 vs. 41 ± 7	63% vs. 68%	35.2 ± 4.8 vs. 33.3 ± 4.2	27 ± 13 vs. 28 ± 11	Not assessed	Not assessed
Herold et al., 2020 (44)	USA	Trial	38.6 ± 12.06 vs. 33.5 ± 11.4	72% vs. 64%	29.31 ± 6.35 Vs. 29.4 ± 6.34	22.1 ± 12.03 Vs. 17.1 ± 9.76	Not assessed	Not assessed
Johansen et al., 2020 (45)	Multi-nations	Trial	29 ± 4.8 vs.27.7 ± 4.1	54% vs. 54%	Not assessed.	21.05 ± 0.90	Not assessed	Not assessed
Kielgast et al., 2011 C-peptide+ve (46)	Multi-nations	Trial	27.0 ± 1.5 32.9 ± 1.7	10% 90%	24.6 ± 0.9 23.1 ± 0.6	3.7 ± 0.8 23.1 ± 1.6	-0.49 ± 0.72 -2.0 ± 0.65	-0.28 ± 0.5 +0.38 ± 0.7
Kielgast et al., 2011 C-peptide-ve (46)	Multi-nations	Trial	35.7 ± 2.2	100%	24.6 ± 0.7 23.1 ± 0.6	17.3 ± 2.5	Not assessed	Not assessed
Kuhadia et al., 2019 (47)	USA	Trial	43 ± 3.3 vs. 50 ± 3	84% vs. 41%	29 ± 3 vs. 28 ± 2	20.3 ± 3 Vs. 19 ± 3	4.5 ± 1 -1.0 ± 0.2 7.2 ± 1 0.77 ± 0.3	18.5 ± 4.33 0.2 ± 0.6 23.5 ± 43 -1.93 ± 0.6
Pozzilli et al., 202 (48)	Italy	Trial	22.3 ± 3.50 Vs. 22.0 ± 3.96	45.7 vs. 40	22.26 ± 3.15 Vs. 22.62 ± 4.35	Not assessed	Not assessed	Not assessed
Zenz et al., 2022 (49)	Austria	Trial	Not assessed	Not assessed	Not assessed	Not assessed	Not assessed	Not assessed
Akturk et al., 2025 (50)	USA	Retrospective	42 ± 8/26	54%	36.7 ± 5.3	Not assessed	1.5 ± 1.7 vs. 1.52 ± 1.66	40.3 ± 16.8 vs. 28.9 ± 6.47
Almohareb et al., 2024 (51)	Saudi Arabia	Retrospective	33 ± 10.1	64.6%	30± 5.7	16.5 ± 7.8	4.0 ± 4.7 Vs. 2.2 ± 3.1	37.6 ± 13.9 vs. 31.1 ± 23.5
Pasqua et al., 2025 (52)	Canada	Trial	45 ± 14	61%	32.2 ± 5.1	28 ± 13	1.4 ± 1.06 vs. 1.5 ± 1.27	37.5 ± 16.3 vs. 29.5 ± 13.8

**Table 2:** Hemoglobin A1c, body weight, type of GLP-1 agonist, and total adverse events among patients on GLP-1 agonists.

Author	HbA1c% at baseline, GLP-1 agonists vs. controls	HbA1c% at end date, GLP-1 agonists vs. controls	Weight at baseline, GLP-1 agonists vs. controls	Weight at end date, GLP-1 agonists vs. controls	Number of patients, GLP-1 agonists vs. controls	Type of GLP-1 agonist/duration	C-peptide, GLP-1 agonists vs. controls
Aherin et al., 2016 (20)	8.67 ± 0.3 8.12 ± 0.4	7.83 ± 0.2 8.11 ± 0.3	83.8 ± 0.82 84.2 ± 0.72	79.34 ± 0.52 84 ± 0.45	203 versus 189	Liraglutide/26 weeks	Not assessed
Mathieu et al., 2016 (21)	8.16 ± 0.75 8.15 ± 0.73	7.69 ± 0.69 7.81 ± 0.33	86.07 ± 17.26 86.4 ± 17.8	82.53 ± 15.90 87.3 ± 16.6	348 versus 348	Liraglutide/52 weeks	Not assessed
Al-Ozairi et al., 2023 (37)	8.1 ± 1.2 8.2 ± 1.3	7.6 ± 0.5 8 ± 0.5	84.8 ± 14.8 82.3 ± 12.8	77.1 ± 7 82.7 ± 17.4	82 versus 80	All/1 year	Not assessed
Dejgaard et al., 2016 (38)	8.7 ± 0.7 8.7 ± 0.7	8.2 ± 0.5 8.4 ± 0.5	93.4 ± 14.12 vs. 94 ± 12.5	86.6 ± 12.77vs.vs. 87.9 ± 10.1	50 versus 50	Liraglutide/24 weeks	Not assessed
Dejgaard et al., 2019 (39)	7.5 ± 0.2 7.9 ± 0.3	8.2 ± 0.5 8.1 ± 0.5	78.7 ± 8.2 77.6 ± 13.6	85 ± 10 Vs. 88 ± 14	24 versus. 24	Liraglutide/24 weeks	Not assessed
Dejgaard et al., 2024 (40)	8.2 ± 2.7 8.6 ± 2.8	6.7 ± 2.6 7.2 ± 2.7	76.6 ± 6.15 73.2 ± 6.29	74.8 ± 6.01 75.4 ± 7.21	31 versus 32	Liraglutide/52 weeks	0.3 ± 0.14 vs. 0.2 ± 0.21
Fandsen et al., 2015 (41)	8.8 ± 0.2 8.7 ± 1	8.2 ± 0.2 8.2 ± 0.2	75.83 ± 2.89 74.89 ± 1.66	72.7 ± 2.9 76.0 6 1.7	18 versus 18	Liraglutide/12 weeks	Not assessed
Ghanim et al., 2020 (42)	7.9 ± 0.19	7.59 ± 0.19	89.6 ± 18.56	85.7 ± 18.56	20 versus 20	Liraglutide/26 weeks	Not assessed
Grag et al., 2025 (43)	7.0 ± 0.9 7.1 ± 1	6.5 ± 0.83/84 6.86 ± 0.91	103.42 ± 18.6 94.8 ± 15.4	76.66 ± 18.4 96.5 ± 15.4	84 versus 38	Tirzepatide/24 weeks	Not assessed
Herold et al., 2020 (44)	7.40 ± 0.81 8 ± 0.49 7.3 ± 0.8 7.55 ± 0.78	7.13 ± 0.48 7.81 ± 0.78 C-peptide +ve 6.79 ± 0.35 Cpeptide –ve 7.25 ± 0.58	83.7 ± 21.7 84.13 ± 22.6	80.78 ± 19.7 84.03 ± 22.6	40 versus 39	Exenatide/24 weeks	Not assessed
Johansen et al., 2020 (45)	8.3 ± 0.8	7.91 ± 1.4	87.7 ± 12.74	81.6 ± 12.74	54 versus 54	Exanatide	Not assessed
Kielgast et al., 2011 C-peptide+ve (46)	6.6 ± 0.3 7.1 ± 0.3	6.4 ± 0.2 6.9 ± 0.2	-2.3 ± 0.3	-2.3 ± 0.3	10 versus 10	Liraglutide/4weeks	0.52 ± 0.11 vs. 0.46 ± 0.08
Kielgast et al., 2011 C-peptide-ve (46)	7.5 ± 0.2 7.1 ± 0.3	7.0 ± 0.1 6.9 ± 0.2	+0.2 ± 0.3 t	+0.2 ± 0.3	9 versus 10	Liraglutide/4weeks	Not assessed
Kuhadia et al., 2019 (47)	10.74 ± 0.96 7.69 ± 0.17	7.4 ± 0.58 7.1 ± 43	71 ± 2 80 ± 6	69 ± 2 79.7 ± 5.5	46 versus 17	Liraglutide/12 weeks	Not assessed
Pozzilli et al., 202 (48)	7.30 ± 1.09 7.27 ± 0.65	-0.59 ± 1.65 -0.73 ± 1.03	66.04 ± 11.87 69.15 ± 13.62	65.27 ± 8.3 68.89 ± 10.88	50 versus. 17	Albiglutide/52 weeks	0.13 ± 0.24 vs. 0.16 ± 0.3
Zenz et al., 2022 (49)	6.9 ± 1.97 6.7 ± 2.33	6.5 ± 2.19 7.1 ± 2.33	70.7 ± 29.84 71.1 ± 30.10	68.6 ± 28.28 71.1 ± 31.75	14 versus. 14	Liraglutide/12weeks	18.98 ± 0.37 vs. 20.99 ± 0.42
Akturk et al., 2025 (50)	7.3 ± 0.7	6.74 ± 0.15	108.1 ± 21.2	96.75 ± 18.97	26	Tirzepatide/32 weeks	Not assessed
Almohareb et al., 2024 (51)	9.0 ± 1.5	8.4 ± 1.3	91.0 ± 17.7	86.9 ± 17.2	144	All/1.5 years	Not assessed
Pasqua et al., 2025 (52)	7.5 ± 0.71	6.8 ± 0.64	91.3 ± 17.4	84.3 ± 17.1	24	Semaglutide/4weeks	

**Table 3:** Total side effects in GLP-1 agonists and controls.

Author	Total side effects of GLP-1 agonists	Total number of patients, GLP-1 agonists	Total side effects, controls	Total number of patients, controls
Aherin et al., 2016 (20)	179	203	160	189
Fandsen et al., 2015 (41)	11	18	9	18
Herold et al., 2020 (44)	38	39	28	35
Kielgast et al., 2011 (46)	10	10	6	10
Pozzilli et al., 202 (48)	41	50	13	17
Pasqua et al., 2025 (52)	304	348	275	348

### Risk of bias assessment

Newcastle Ottawa Scale risk of bias, and Cochrane Risk of Bias assessed the risk of bias of the included studies (35, 36). The risk of bias of the observational studies ranged from 7 to 9. **Tables 4**, **5**. The grade of evidence was assessed by Grading of Recommendations Assessment, Development, and Evaluation (GRADE) **Table 6**.

**Table 4:** The Cochrane risk of bias assessment tool of the included studies.

Author	Random sequence generation bias.	Allocation concealment bias	Blinding of participants and personnel.	Blinding of outcome assessment	Incomplete outcome data	Selective reporting	Other bias
Aherin et al., 2016 (20)	Low	Low	Low	Unclear	low	unclear	low
Mathieu et al., 2016 (21)	Low	Low	Unclear	Unclear	Unclear	Low	Unclear
Dejgaard et al., 2016 (38)	Low	Low	Low	Low	Low	Low	Low
Dejgaard et al., 2019 (39)	Low	Low	Low	Low	Low	Low	Low
Dejgaard et al., 2024 (40)	Low	Low	Low	Low	Low	Low	Low
Frandsen et al., 2015 (41)	Low	Low	Low	Low	Low	Low	Low
Ghanim et al., 2020 (42)	Low	Low	Low	High	High	High	High
Herold et al., 2020 (44)	Low	Low	Low	Unclear	Unclear	low	low
Johansen et al., 2020 (45)	Low	Low	Low	Low	Low	Low	Low
Kielgast et al., 2011 (46)	Low	Low	Low	Unclear	Unclear	low	low
Kuhadiya et al., 2016 (47)	Low	Low	Low	Unclear	Low	Low	Unclear
Pozzilli et al., 2020 (48)	Low	Low	Low	Low	Low	Low	Low
Zenz et al., 2022 (49)	Low	Low	Low	Low	Low	Low	Low
Pasqua et al., 2025 (52)	Low	Low	Unclear	Unclear	Low	Low	Low

**Table 5:** Newcastle Ottawa scale risk of bias of the included observational studies.

Author	Selection bias	Comparability bias	Outcome	Total score
Al-Ozairi et al., 2023 (37)	4	2	3	9
Grag et al., 2025 (43)	4	1	3	8
Akturk et al., 2025 (50)	3	1	3	7
Almohareb et al., 2024 (51)	4	2	3	9

**Table 6:** Analysis of the quality of evidence by grading of recommendations assessment, development, and evaluation (GRADE).

Outcome	Studies	Study design	Risk of bias	Inconsistency	Indirectness	Imprecision	Other considerations	Certainty of evidence
Body weight	15	Trials	serious	Serious (*I^2^* = 100%)	Not serious	Not serious	None	Very low
HbA1c	15	Trials	Serious	Serious (*I^2^* = 100%)	Not serious	Not serious	None	Very low
Time in hypoglycemia	7	Two observational and 5trials	serious	Serious (*I^2^* = 98%)	Not serious	Not serious	None	Very low
Time in hyperglycemia	7	Two observational and 5 trials	Serious	Serious (*I^2^* = 97%)	Not serious	Not serious	None	Very low
Stimulated C-peptide	4	Trials	serious	Serious (*I^2^* = 93%)	Not serious	Not serious	None	Very low
Total adverse events	6	Trials	Serious	Not serious (*I^2^* = 3%)	Not serious	Not serious	None	Very low

### Statistical analysis

The most recent version of the RevMan system (version 5.4.1, United Kingdom) was used for data analysis. We included 18 studies. The continuous data for weight, HbA1c, time spent in hypoglycemia and hyperglycemia, and C-peptide levels were entered manually, and the random effect was used for substantial heterogeneity. The continuous data were presented as a standard mean difference, using a Forest plot at a 95% confidence interval, and Funnel plots were generated for heterogeneity. The Chi-Square test, the weighted average effect size (Z), and the standard difference were applied. The Egger,s regression test was not conducted due to the extreme heterogeneity (I^2^ = 100%). A subgroup analysis was conducted in which references were removed one by one to locate heterogeneity. In addition, we conducted a subgroup analysis including only obese patients. A P-value of <0.05 was considered significant.

## Results

### Characteristics of the included studies

There were 18 studies (20, 21, 37–52), 15 were trials, and 4 were retrospective studies. Nine studies were from Europe, five studies were published in the United States of America, 2 studies were conducted in Asia, two were multinational, and one was published in Canada. The age of the patient ranged from 22.26 ± 3.15 to 50 ± 14.

Years (10-100% women), the duration of diabetes ranged from 3.7 ± 0.8 to 32 ± 11 years, the body mass index ranged from 22.0 ± 3.96 to 50 ± 3 (the majority were obese=12 studies, overweight=3 studies, normal weight=1 study, and the BMI was not reported in 2 studies), the duration of studies ranged from 4 weeks to one year, and HbA1c ranged from 6.7 ± 2.33 to 10.74 ± 0.96. In this study, liraglutide was the most common GLP-1 agonist used, followed by exenatide (2 studies) and semaglutide in three studies. **Tables 1**, **2**. The risk of bias was assessed by the Newcastle Ottawa Scale for retrospective studies and the Cochrane Risk of Bias for clinical trials **Table 3**.

**Figure 2:**
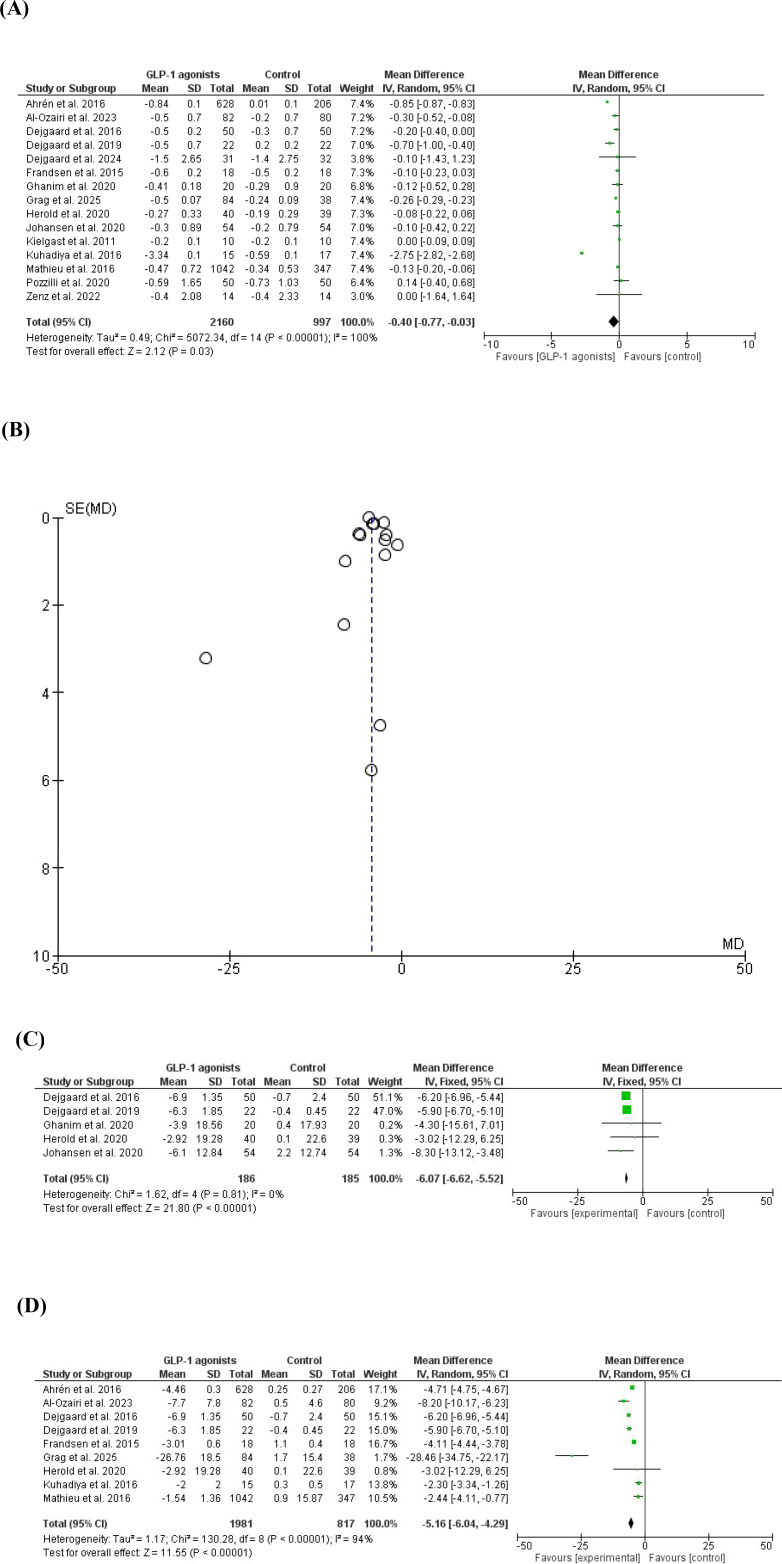
**(A)** Weight reduction in patients with type 1 diabetes mellitus (forest plot). **(B)** Weight reduction in patients with type 1 diabetes mellitus (funnel plot). **(C)** Weight reduction in patients with type 1 diabetes mellitus (forest plot, no heterogeneity). **(D)** Weight reduction in patients with type 1 diabetes mellitus (forest plot, including only obese patients).

A higher weight reduction was found in GLP-1 agonists cases compared to placebo/control in which 15 studies (20, 21, 37–49) with 3157 patients were included, MD=-4.28, 95% *CI*, -5.06--3.49, a significant heterogeneity was observed, *I^2^* = 97%, P-value for heterogeneity <0.001 Chi-square=488.70, P-value for overall effect < 00.1, and Z = 10.70. A subgroup analysis was conducted to remove studies with high contribution to heterogeneity with higher weight reduction in the GLP-1 agonists group compared to control, MD=-6.07, 95% *CI*, -6.62--5.52, no significant heterogeneity was observed, *I^2^* = 0%, P-value for heterogeneity, 0.81 Chi-square=1.62, P-value for overall effect < 00.1, and Z = 21.80. Additionally, a subgroup analysis was conducted to include only obese patients, weight reduction was higher in GLP-1 agonists arm, MD=-5.16, 95% *CI*, -6.04--4.29, a significant heterogeneity was observed, *I^2^* = 94%, P-value for heterogeneity < 0.001 Chi-square=130.28, P-value for overall effect < 00.1, and Z = 11.55 **Figures 2A–D**.

The HbA1c was reduced in 15 studies (20, 21, 37–49), MD -0.4, 95% *CI*, -0.77--0.03; a significant heterogeneity was observed, *I^2^* = 100%, P-value for heterogeneity <0.001, Chi-square=5072.34, P-value for overall effect, 0.03, and Z = 2.12. However, the HbA1c was marginally lower in GLP-1 agonists compared to control when removing studies with high contribution to heterogeneity, MD -0.6, 95% *CI*, -0.12-0.00; no significant heterogeneity was observed, *I^2^* = 0%, P-value for heterogeneity, 0.78, Chi-square=4.76, P-value for overall effect, 0.06, and Z = 1.89.

**Figure 3:**
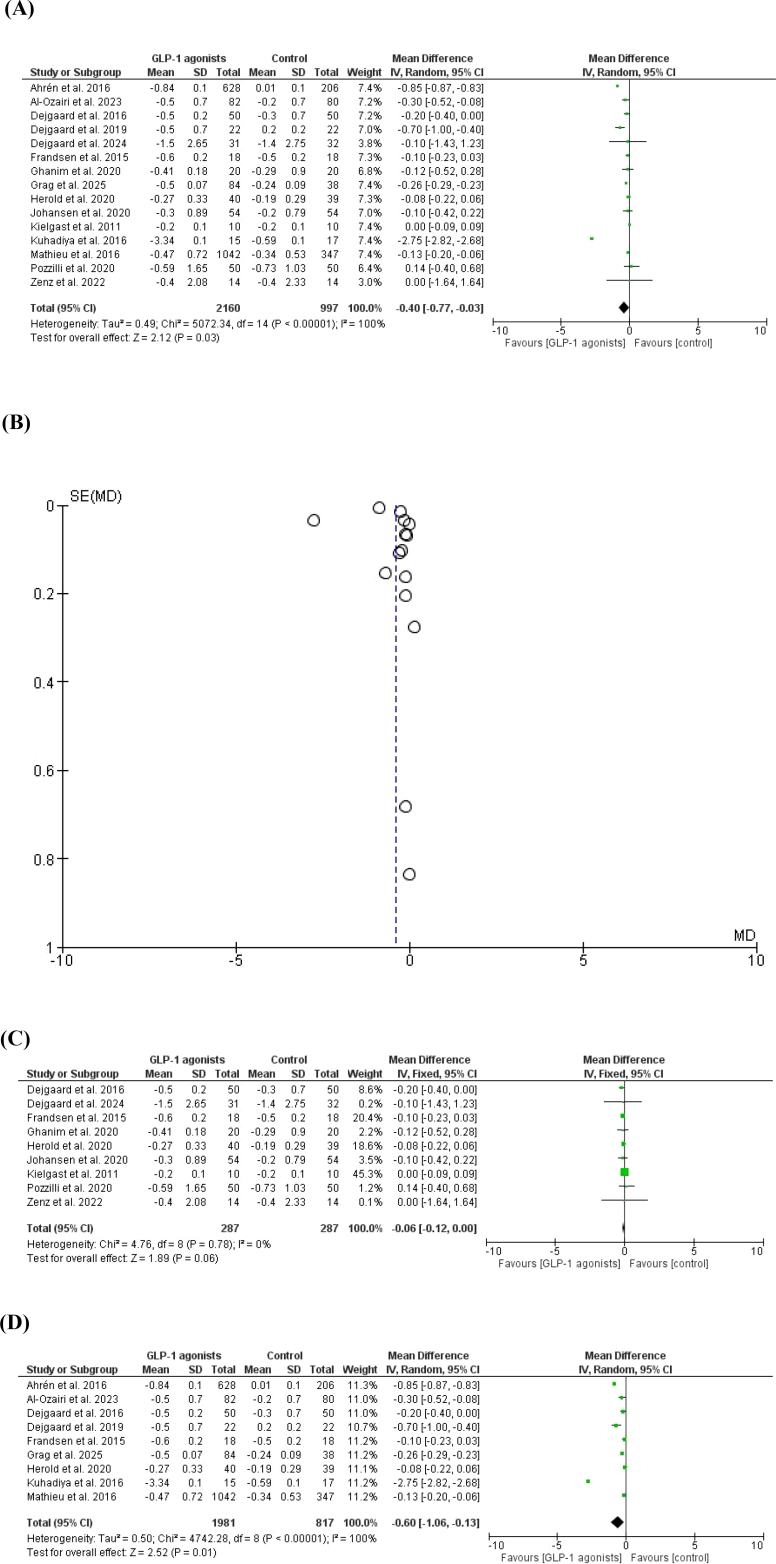
**(A)** HbA1c reduction in cases versus placebo/control studies (forest plot). **(B)** HbA1c reduction in cases versus placebo/control studies (funnel plot). **(C)** HbA1c reduction in cases versus placebo/control studies (forest plot, no heterogeneity). **(D)** HbA1c reduction in cases versus placebo/control studies (forest plot, no heterogeneity).

GLP-1 agonists showed a higher reduction of HbA1c compared to placebo when including only obese patients, MD -0.60, 95% *CI*, -1.06--0.13; a significant heterogeneity was observed, *I^2^* = 100%, P-value for heterogeneity< 0.001, Chi-square=4742.28, P-value for overall effect< 0.001, and Z = 2.52 **Figures 3A–D**.

**Figure 4:**
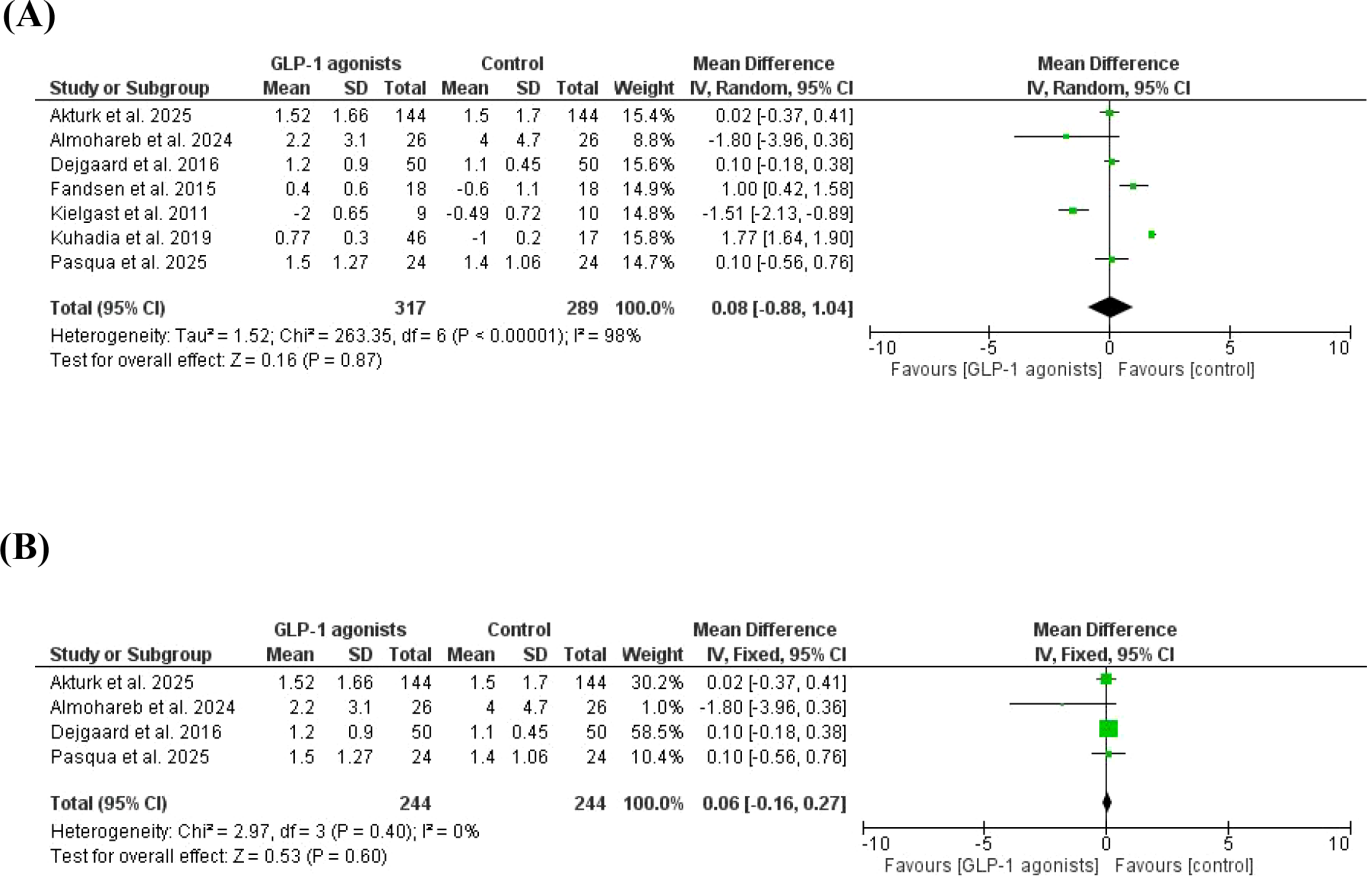
**(A)** Time spent in hypoglycemia, GLP-1 agonist at baseline, and after GLP-1 agonists. **(B)** Time spent in hypoglycemia, GLP-1 agonist at baseline, and after GLP-1 agonists (no heterogeneity).

The total time spent in hypoglycemia was not different between case/control (38, 41, 46, 47, 50–52), MD = 0.08, 95% *CI*, -0.88-1.04. A significant heterogeneity was observed, *I^2^* = 98%, P-value for heterogeneity, < 0.001, Chi-square=263.35, P-value for overall effect, 0.87, and Z = 0.16. The results were not different in a subgroup analysis after removing studies with high contribution to heterogeneity, MD = 0.06, 95% *CI*, -0.16-0.27. No significant heterogeneity was observed, *I^2^* = 0%, P-value for heterogeneity, 0.40, Chi-square=2.97, P-value for overall effect, 0.60, and Z = 0.53 **Figures 4A, B**.

**Figure 5:**
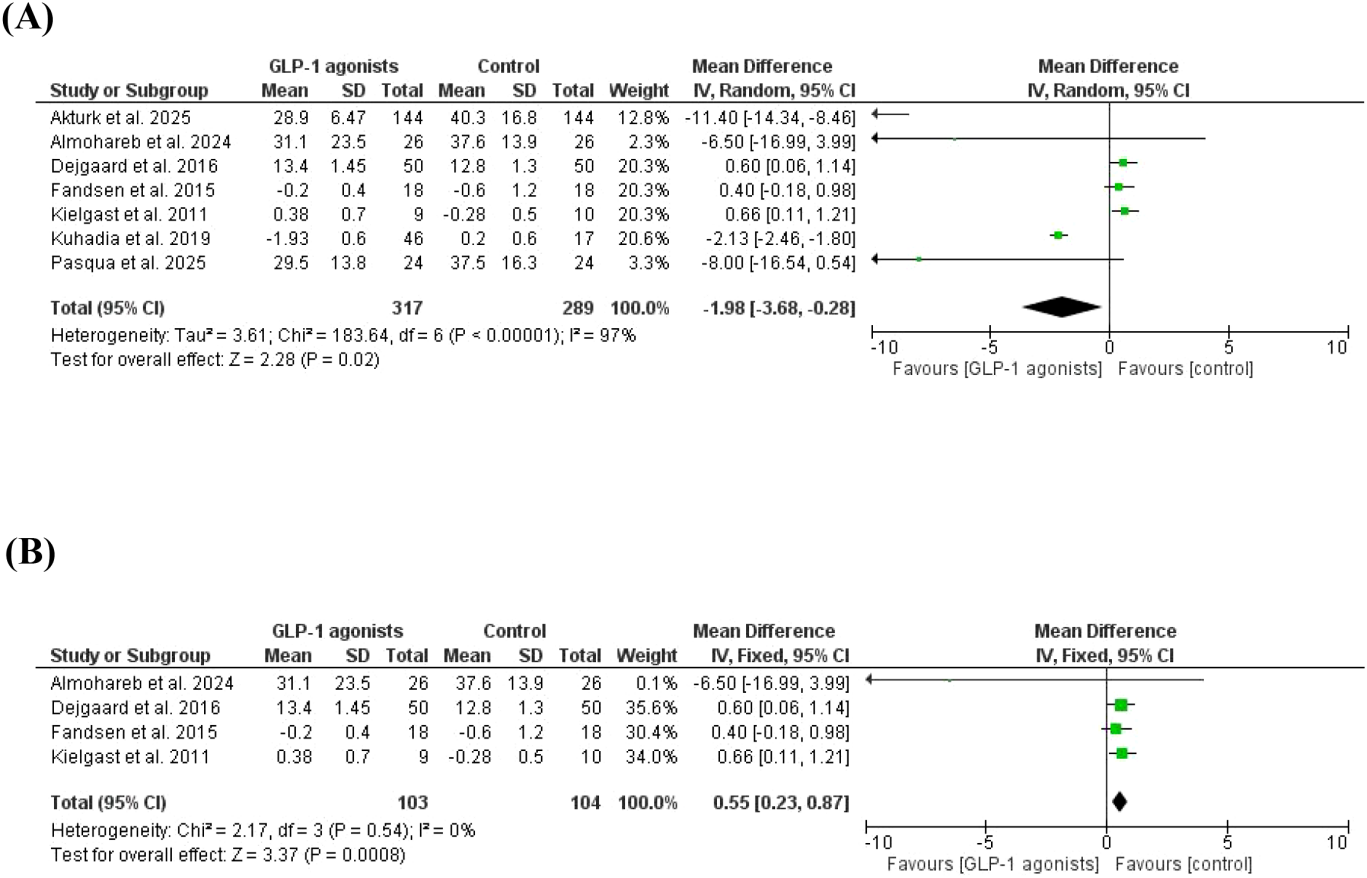
**(A)** Time spent in hyperglycemia, GLP-1 agonist at baseline, and after GLP-1 agonists. **(B)** Time spent in hyperglycemia, GLP-1 agonist at baseline, and after GLP-1 agonists (no heterogeneity).

The time spent in hyperglycemia was lower in patients on GLP-1 agonists versus controls (38, 41, 46, 47, 50–52), MD=-1.98, 95% *CI*, -3.68--0.28, a significant heterogeneity was observed, *I^2^* = 97%, P-value for heterogeneity, < 0.001, Chi-square=183.64, P-value for overall effect, 0.02, and Z = 2.28. The results were not different in a subgroup analysis after removing studies with a high contribution to heterogeneity, MD = 0.55, 95% *CI*, 0.23-0.28. No significant heterogeneity was observed, *I^2^* = 0%, P-value for heterogeneity, 0.54, Chi-square=2.17, P-value for overall effect, 0.0008, and Z = 3.37 **Figures 5A, B**.

**Figure 6:**
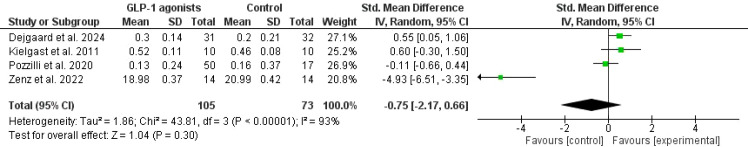
Stimulated C-peptide among patients on GLP-1 agonists.

The maximum stimulated C-peptide was not different in patients on GLP-1 agonists and placebo (40, 46, 48, 49), MD=-0.75, 95% *CI*, - 2.17-0.66. A significant heterogeneity was observed, *I^2^* = 0.0%, P-value for heterogeneity, < 0.001, Chi-square=43.81, P-value for overall effect, 0.30, and Z = 1.04 **Figure 6**.

**Figure 7:**
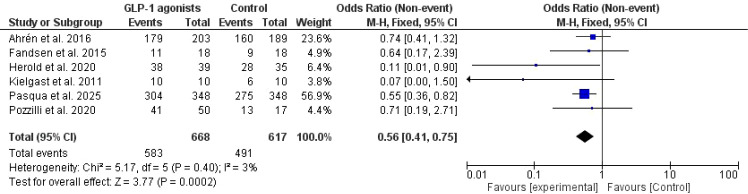
Total adverse events in GLP-1 agonists and controls.

The total adverse events were higher in patients on GLP-1 agonists versus controls (20, 41, 44, 46, 48, 52), Odd ratios=0.56, 95% *CI*, 0.41-0.75, no significant heterogeneity was observed, *I^2^* = 3%, P-value for heterogeneity, 0.40, Chi-square=5.17, P-value for overall effect, 0.0002, and Z = 3.77 **Figure 7**.

## Discussion

In this meta-analysis, a significant weight reduction was found following GLP-1 agonist use, MD=-4.28, 95% *CI*, -5.06--3.49; the results were significant after removing studies with high heterogeneity, MD=-6.07, 95% *CI*, -6.62--5.52. GLP-1 agonists significantly reduced HbA1c, MD=-4.28, 95% *CI*, -5.06--3.49; the results were marginally significant after removing studies with high heterogeneity, MD=-0.6, 95% *CI*, -0.12-0.00. Karakasis et al. (31) included only 6 trials with 378 patients and found significant HbA1c reduction, lower time above range, and higher time below range. Our findings supported their findings regarding HbA1c reduction and the time spent in hyperglycemia. However, we found no differences regarding time below the range. The discrepancy could be explained by the small number of patients in Karakasis et al. (31). In addition, they included Giang et al. (53), who examined glucose excursion in response to hypoglycemia. Our findings were similar to Park et al. (32) regarding weight loss, but they included Giang et al. (53), who assessed glucose excursion in response to hypoglycemia. In addition, they included a correspondence that limited their results (54). The authors found similar weight reduction in C-peptide positive and C-peptide negative patients; we found no difference between the maximum stimulated C-peptide in patients on GLP-1 agonists and placebo, SMD=-0.75, 95% *CI*, - 2.17-0.66. GLP-1 agonists fail to significantly increase C-peptide in type 1 diabetes because the disease lacks functional β-cells particularly with long diabetes duration, and GLP-1 therapy does not restore or protect β-cell mass. Our findings are similar to Von Herrath et al. (55), who found liraglutide monotherapy had minimal effect on C-peptide secretion. The above results imply that GLP-1 agonists are beneficial for weight and HbA1c reduction. The HbA1c reduction could be due to weight reduction and a direct effect on the β-cell of the pancreas. Other mechanisms of GLP-1 agonists on HbA1c could be the downregulation of glucagon. T1DM is characterized by hyperglucagonemia, glycogenolysis, and gluconeogenesis by the liver and kidneys to maintain blood glucose in the fasting state (56, 57). Therefore, downregulation of glucagon by GLP-1 agonists helps to maintain blood glucose (27). Our findings supported the above observation. Previous meta-analyses observed a high rate of hypoglycemia in patients on GLP-1agonists (58, 59), in contradiction to the current results, in which the time spent in hypoglycemia was not affected by GLP-1 agonist use. Wang et al. (34) concluded the positive effects of GLP-1 agonists on insulin on weight and HbA1c. However, their results were limited by including only 7 studies (one included DPP-4 inhibitors, one poster presentation, and one included daclizumab, which could affect beta cell function (60–63).

Importantly, our findings found weight and HbA1c reduction in patients with T1DM with a similar effect on hypoglycemia. Therefore, GLP-1 agonists counteract insulin-induced weight gain. However, the majority of patients were obese with long duration of T1DM; the appropriate timing of GLP-1 agonists use as adjuvant therapy is to be determined; a piece of evidence suggested that early introduction of combination therapy with insulin preserves beta cell function (55).

Our meta-analysis is novel because, we included the largest up to the date number of studies, and assessed time spent in hyperglycemia and hypoglycemia. In addition, we assessed the maximum stimulated C-peptide. Furthermore, we included long-acting GLP-1 agonists (semaglutide, albiglutide, and tirzepatide). Importantly we excluded correspondence, poster presentation, response to glucose excursion, and studies comparing GLP-1 agonists with daclizumab.

### The study limitation

The study limitations are the high heterogeneity observed and the small number of studies assessing the time spent in hypoglycemia and hyperglycemia. In addition, we could not perform relevant subgroup analyses according to baseline characteristics of interest, including obesity-related comorbidities, and level of glycaemia. A major limitation of this study is that the majority of the included studies assessed obese/overweight patients. Therefore, the current results cannot be generalized to all patients with T1DM. Importantly, the duration of some of the included studies might not be enough to achieve glycemic steady state. A major limitation of this study is that we could not assess for major variables including ketosis and diabetic ketoacidosis. Another important limitation is the small number assessing hypoglycemia and hyperglycemia.

## Conclusion

GLP-1 agonists reduced weight, HbA1c, and time in hyperglycemia significantly compared to controls at the cost of total side effects. The stimulated C-peptide and hypoglycemia were not different between the two groups. The endpoints were assessed in obese patients with a long duration of diabetes. Larger controlled trials including normal weight patients with type 1 diabetes, and newly diagnosed patients with preserved beta cell function are recommended.

## Data Availability

The original contributions presented in the study are included in the article/supplementary material. Further inquiries can be directed to the corresponding author.
